# Risk-based indicators of Canadians’ exposures to environmental carcinogens

**DOI:** 10.1186/1476-069X-12-15

**Published:** 2013-02-12

**Authors:** Eleanor Setton, Perry Hystad, Karla Poplawski, Roslyn Cheasley, Alejandro Cervantes-Larios, C Peter Keller, Paul A Demers

**Affiliations:** 1 Department of Geography, University of Victoria, Victoria, BC, Canada; 2 School of Population and Public Health, University of British Columbia, Vancouver, BC, Canada; 3 Department of Geography, University of British Columbia, Vancouver, BC, Canada; 4 Occupational Cancer Research Centre, Cancer Care Ontario, Ontario, Canada

**Keywords:** Canada, Risk, Carcinogens, Cancer, Environment, Air, Food, Dust, Water, Beverages, Policy

## Abstract

**Background:**

Tools for estimating population exposures to environmental carcinogens are required to support evidence-based policies to reduce chronic exposures and associated cancers. Our objective was to develop indicators of population exposure to selected environmental carcinogens that can be easily updated over time, and allow comparisons and prioritization between different carcinogens and exposure pathways.

**Methods:**

We employed a risk assessment-based approach to produce screening-level estimates of lifetime excess cancer risk for selected substances listed as known carcinogens by the International Agency for Research on Cancer. Estimates of lifetime average daily intake were calculated using population characteristics combined with concentrations (circa 2006) in outdoor air, indoor air, dust, drinking water, and food and beverages from existing monitoring databases or comprehensive literature reviews. Intake estimates were then multiplied by cancer potency factors from Health Canada, the United States Environmental Protection Agency, and the California Office of Environmental Health Hazard Assessment to estimate lifetime excess cancer risks associated with each substance and exposure pathway. Lifetime excess cancer risks in excess of 1 per million people are identified as potential priorities for further attention.

**Results:**

Based on data representing average conditions circa 2006, a total of 18 carcinogen-exposure pathways had potential lifetime excess cancer risks greater than 1 per million, based on varying data quality. Carcinogens with moderate to high data quality and lifetime excess cancer risk greater than 1 per million included benzene, 1,3-butadiene and radon in outdoor air; benzene and radon in indoor air; and arsenic and hexavalent chromium in drinking water. Important data gaps were identified for asbestos, hexavalent chromium and diesel exhaust in outdoor and indoor air, while little data were available to assess risk for substances in dust, food and beverages.

**Conclusions:**

The ability to track changes in potential population exposures to environmental carcinogens over time, as well as to compare between different substances and exposure pathways, is necessary to support comprehensive, evidence-based prevention policy. We used estimates of lifetime excess cancer risk as indicators that, although based on a number of simplifying assumptions, help to identify important data gaps and prioritize more detailed data collection and exposure assessment needs.

## Background

The International Agency for Research on Cancer has identified one hundred and nine environmental factors that can increase cancer risk in humans, including a range of chemicals and complex mixtures, exposure circumstances (i.e., certain occupations), physical agents (i.e., solar radiation), biological agents (i.e., certain viruses) and lifestyle factors (i.e., tobacco smoking) [1,2]. Estimates of the proportion of cancers due to environmental exposures (defined in this article as pollution or contamination) range from <1% to 29% [3-5], and as these exposures are typically considered to be modifiable, reducing or eliminating exposures presents an opportunity to decrease future cancer incidence. It has further been suggested that the contribution of exposure to low levels of carcinogens in the environment to overall cancer burden has been underestimated, and that a new prevention paradigm is needed that recognizes cancer is caused by multiple interacting factors, and therefore we should limit exposures to avoidable environmental and occupational carcinogens, in combination with other factors such as diet and lifestyle [6].

In 2007, in response to recommendations from its National Committee on Environmental and Occupational Exposures [7] and external organizations, such as the Canadian Cancer Society, the Canadian Partnership against Cancer (CPAC) funded the CARcinogen EXposure (CAREX) Canada project as part of its primary prevention efforts. The goal of CAREX Canada is to develop and implement exposure surveillance methods for a range of known or suspected carcinogens. CAREX Canada includes an occupational component that builds off the original CAREX project developed by IARC and the Finnish Institute for Occupational Health [8], and a non-occupational component, which we identify as ‘environmental’. For some key lifestyle risk factors (e.g. diet, physical activity and smoking) estimates of prevalence and trends over time in the general population exist in Canada, for example through national health surveys [9]. For other risk factors, however, such as exposures to chemical and physical agents, these fundamental aspects of the cancer control spectrum are not well developed [10]. The CAREX Canada environmental indicators therefore focus on carcinogens present in outdoor air, indoor air, indoor dust, drinking water, and foods and beverages (note: exposures via dermal absorption are not included due to a pervasive lack of data on concentration and product use/frequency of exposure levels). The scope of CAREX Canada does not include the collection of primary data. Efforts therefore focus on using existing data only. This distinguishes CAREX Canada from exposure surveillance programs that take an active individual monitoring approach, for example the National Dose Registry [11].

In keeping with the population-level focus of the CAREX mandate, we developed three guiding principles for developing indicators for surveillance of exposures to environmental carcinogens in Canada: (1) indicators should be based on regularly collected and available data, supporting ongoing surveillance over time; (2) indicators should consider a range of environmental media, including outdoor air, indoor air or dust, drinking water, and food and beverages; and, (3) indicators should allow for comparisons among substances, exposure pathways, populations and geographic locations in order to support prioritization and targeted prevention efforts.

**Figure 1:**
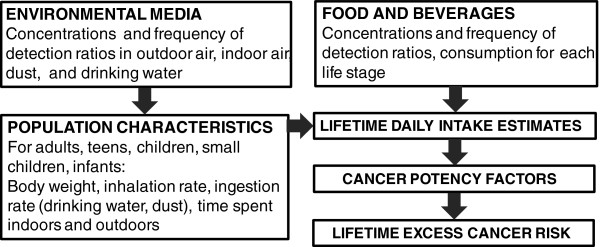
Framework for calculating lifetime potential excess cancer risk for the Canadian population associated with different carcinogens and exposure pathways.

We adopted a risk-based approach, requiring the calculation of lifetime average daily intake by major exposure routes, and the associated potential lifetime excess cancer risk (LECR). A risk-based approach was chosen to allow comparisons between substances and exposure pathways and to provide an indicator that is readily interpretable by a wide range of stakeholders. Figure 1 provides a simplified schematic of the required input data and typical sources, which are further described below.

Other methods for risk-based ranking also exist. Our approach of estimating average daily intake is similar to the assessments conducted under the Canadian Environmental Protection Act (CEPA); however, the CEPA daily intake estimates are not converted to lifetime excess cancer risk, but are used to develop a “priority for action” ranking when the substance is considered to be carcinogenic [12]. We chose to use potential excess lifetime risk as it seems more intuitively understandable, and under the right circumstances (i.e., same target organ and form of cancer), risk estimates may be added to reflect cumulative exposure risks [13].

The limitations of the cancer potency factors employed here include the extrapolation of experimental results observed in animals exposed to high doses to humans generally exposed to low doses, the assumption of a linear relationship between dose and response, and the assumption of no effect threshold. Ongoing research continually provides new information on the validity of these assumptions [14-16]. For example, there is increasing evidence that the dose–response relationship for a number of substances (including diesel engine exhaust, formaldehyde, lead, nickel and TCDD), may be hormetic, that is, “u- or j-shaped”, indicating different effects depending on the exposure level [17]. An alternative to using cancer potency factors is to use concentration-response (CR) functions from published human epidemiology studies. These would typically have smaller uncertainties than cancer potency factors based on interspecies extrapolation; however, CR functions have been developed for relatively few substances and exposure routes (primarily criteria air pollutants in air), which would have greatly limited comparison and prioritization of the range of environmental carcinogens present in Canada.

Individual indicators for each substance and exposure pathway combination are presented here; however it is critical to acknowledge that the interactions among substances and exposures via different pathways are complex. The true relationships between the development of cancer and concurrent exposures to a wide variety of chemical and natural carcinogens in conjunction with the influences of lifestyle factors throughout an individual’s lifetime are not (and may never be) well understood [6,18,19].

The indicators do not represent the prevalence of exposure in the Canadian population. Nationally representative, multi-substance and multi-exposure pathway monitoring programs would be required to establish prevalence, and these types of initiatives do not exist in Canada now or in the foreseeable future. We propose the indicators developed here are useful as a starting point to help guide more focused data gathering and exposure assessment work where identified gaps exist, and to support regulatory progress and public outreach when reasonable evidence exists that known carcinogens are present in the Canadian setting at levels sufficient to be of potential concern.

## Methods

The underlying calculations conducted in Figure 1 are based on standard risk assessment methods [13,20,21] and assumptions (Table 1). For a given substance and life-stage, daily intake in micrograms per kilogram of bodyweight is calculated as follows for each exposure pathway (outdoor air, indoor air, dust, drinking water) with the exception of food/beverages:

(1) $$\text{DI}=\left(\left(C_{P}*{\text{EF}}_{P}\right)*I_{R}*T_{L}\right)/\text{BW}$$

where:

DI = daily intake

C_P_ = concentration in pathway (outdoor air, indoor air, dust, soil, drinking water)

EF_P_ = for the specific pathway, the exposure frequency

I_R_ = daily intake rate via inhalation or ingestion

T_L_ = percent of day spent indoor or outdoor, applied to outdoor air, indoor air, dust and soil only

BW = bodyweight for given life-stage

**Table 1:** Standard physical characteristics

**Characteristic**	**Units**	**Adult**	**Teen**	**Child**	**Small Child**	**Infant**
Age	years	20 to 70	12 to 19	5 to 11	0.5 to 4	0 to 0.5
Bodyweight	kilograms	70	57	27	13	6
Breathing	cubic metres per day	23	21	12	5	2
Drinking water	litres per day	1.5	1.3	0.9	0.8	0.75
Dust ingestion	grams per day	0.02	0.02	0.035	0.05	0.035
Time outdoor	% of 24 hours	6.25	6.25	8.2	8.2	8.2
Time indoors	% of 24 hours	93.75	93.75	91.8	91.8	91.8

Daily intake via ingestion of food and beverages is calculated the same way (excluding T_L_) for each specific food. These are then summed for each of seven groups – meat, seafood, fruit, vegetables, dairy and eggs, grains and nuts (including breads), and beverages.

Exposure frequency (EF_p_) is included as a means of modifying estimates according to how often exposure is expected to occur. This parameter can be used in several ways. First, a value of 1 (i.e., 100 percent) could be used to reflect a carcinogen that is pervasive and therefore all members of a population are expected to be exposed (for example, outdoor air pollution). A value of 1 could also be used to represent a contamination scenario in which all of the intake amount has detectable levels, but the resulting intake and risk estimates apply only to exposed populations (for example, only those people drinking well water contaminated with benzene from leaking underground fuel tanks). Secondly, including a percent value of less than 1, for example 0.3 implies that exposure occurs only in 30 percent of the intake amount(for example, the substance has been detected in only 30 percent of samples tested).

Given the calculated daily average intake for each lifestage, substance and exposure pathway combination, a single estimate of intake by exposure pathway over an entire 70 year life is calculated by weighting each life-stage specific intake level by the amount of time spent in each life-stage, then summing:

(2) $${\text{LSW}}_{\text{DI}}=S\left({\text{LS}}_{i}*T_{i}\right)$$

where

LSW_DI_ = life-stage weighted daily intake

LS_i_ = daily intake for life-stage i

T_i_ = percent of time spent in life-stage i, expressed as time in life-stage i/ total lifetime

Given an estimated lifetime average daily intake in mg/kg of bodyweight, cancer potency factors are then applied to estimate the associated LECR:

(3) LifetimeExcessCancerRisk=AverageDailyIntake*CancerPotencyFactor

This approach applies to many known and suspected carcinogens; however, it is important to note that for radon, lifetime excess cancer risk is calculated using total lifetime dose [22] not lifetime average daily intake. In radon’s case, the above equations were modified as necessary. Similarly, for asbestos, the average hourly concentration over an entire lifetime is treated as the representative intake (dose) and LECR is calculated by multiplying the lifetime average hourly concentration by an inhalation unit risk factor [23].

Availability and quality of the input data varied widely depending on the substance and exposure pathway considered. Existing, readily available and ongoing national databases were used whenever possible, but we also had to rely on data from government reports and peer-reviewed studies reporting measured concentrations. We used data only from studies conducted in Canada, the US and northern European countries with data collected in 2000 or more recently.

Outdoor air concentrations are based either on quality-assured data from the Canadian National Air Pollution Surveillance (NAPS) monitoring system for 2006 [24] or peer-reviewed literature and government reports published since 2000. Typically, data from the NAPS monitoring system are of high quality in terms of instruments used and their calibration, regularity of the sampling intervals over an entire year, and the geographic distribution of stations across Canada [25]. Notably, data for radon and asbestos came from government reports and peer-reviewed literature. Radon has been measured extensively across Canada using accepted monitoring protocols [26]. Asbestos, however, is not regularly measured in outdoor air in Canada, and different methods exist for measuring levels which can produce substantially different results [27], presenting a potentially important data gap.

Indoor air and dust concentrations are based on data published in peer-reviewed literature since 2000. In general, other than for radon, benzene and formaldehyde in indoor air, we found few studies reporting measured levels of our selected carcinogens in these exposure pathways. Sample sizes were relatively small, and studies often focused in one geographic location. For dust, analytical methods varied (we used only data analyzed using inductively coupled plasma mass spectrometry, the most accurate currently available) and results were often presented as volume per square centimeter cm^2^, rather than in micrograms per gram μg/g, and so we were not able to include these in our estimates.

Drinking water data are from the Ontario Drinking Water Surveillance Program (DWSP) for 2006 for distribution systems (not raw water or treated water at plant) [28]. In addition, a review of published literature and government reports on drinking water for Canada was conducted, and levels compared to those from DWSP. In Canada, drinking water testing is conducted by local municipal governments, and results are not typically available in an easily accessible form, like the Ontario DWSP. Private wells are tested only by individuals, and the lack of data for these Canadians is a significant gap, particularly with respect to arsenic in drinking water.

The list of foods included in this study was derived from the Canada Food Stats database [29]. Consumption levels for adults, teens, children, small children and infants were based on levels specific to each life stage from the Nutrition Canada Survey [30] when available, otherwise per capita loss-adjusted consumption from the Canada Food Stats database were used to represent adult consumption, and reduced in proportion to bodyweight for other life stages. Concentrations in foods are primarily from the Canadian Food Inspection Agency Chemical Residues in Food reports [31], the United States Total Diet Study results [32] and the Dietary Exposure Potential Model, which contains concentration data from numerous United States studies conducted prior to 2003 [33]. Our search for Canadian food and beverage data revealed substantial data gaps. Importantly, no databases were found that included both consumption levels and concentration levels, and we encountered difficulties in matching the foods listed between each different database [34]. As well, consumption data are based either on 1) a national 24 hour dietary recall survey conducted in the early 1970s (still the most comprehensive survey done for Canadians) [30] or 2) per capita estimates based on amount of food available nationally [29]. We do not know how well these data sources represent the average Canadian diet now or over the long term.

Varying data availability limits the representativeness of some of the indicators. We focused on creating estimates of mean measured levels as inputs for the indicators, in the absence of data that would support the development of valid exposure distributions in the Canadian population. Qualitative assessments of how well the data used represent the ‘average’ for Canadians for each substance/exposure pathway are provided with the results and summarized in Table 2. This approach is consistent with recommendations for screening-level assessments [35,36]. More details on the basis for assigning data quality ranks (gap, very low, low, moderate and high) shown in the following results are available on the CAREX Canada website [37], as are documentation and citations for all data sources and levels used to calculate the LECRs reported here.

**Table 2:** Summary of Canadian indicators of lifetime excess cancer risk for known carcinogens and each relevant exposure pathway

**Carcinogen**	**Average Concentration**	**Data Quality**	**Lifetime Excess Cancer Risk Estimates**		
			**Average concentration andCPF**^**1**^**from:**		
			**CA**^**2**^	**HC**^**3**^	**EPA**^**4**^
**Indoor Air**					
Arsenic and compounds	---	gap	-	-	-
Asbestos	8.5x10^-5^ f/ml	very low	10.8	-	1.3
Benzene	2.4 μg/m^3^	moderate	78.0	11.4	21.1
Benzo[a]pyrene	1.9 x10^-4^ μg/m^3^	very low	0.2	< 0.1	-
1,3-Butadiene	0.12 μg/m^3^	low	23.4	-	3.9
Cadmium and compounds	---	gap	-	-	-
Chromium (hexavalent)	---	gap	-	-	-
Diesel engine exhaust	0.84 μg/m^3^	very low	300.2	-	-
Formaldehyde	33.3 μg/m^3^	low/moderate	227.2	-	486.8
Nickel and compounds	8.5 x10^-4^ μg/m^3^	low	0.3	0.9	-
Radon	100 Bq/m^3^	moderate/ high	-	-	23,655.0
TCDD	---	gap	-	-	-
**Outdoor Air**					
Arsenic and compounds	4.3x10^-4^ μg/m^3^	moderate	0.1	0.3	0.2
Asbestos	2.0 x10^-5^ f/ml	very low	2.5	-	0.3
Benzene	0.86 μg/m^3^	high	2.0	0.3	0.5
Benzo[a]pyrene	1.4 x10^-4^ μg/m^3^	moderate	<0.1	< 0.1	-
1,3-Butadiene	0.096 μg/m^3^	high	1.3	-	0.2
Cadmium and compounds	1.2 x10^-4^ μg/m^3^	moderate	0.1	0.1	<0.1
Chromium (hexavalent)	1.6 x10^-5^ μg/m^3^	low	<0.1	0.1	<0.1
Diesel engine exhaust	1.4 μg/m^3^	very low	35.6	-	-
Formaldehyde	1.6 μg/m^3^	moderate	0.8	-	1.7
Nickel and compounds	7.0 x10^-4^ μg/m^3^	moderate	<0.1	0.1	-
Radon	24 Bq/m^3^	moderate	-	-	371.0
TCDD	9.7 x10^-10^ μg/m^3^	moderate	<0.1	-	<0.1
**Drinking Water**					
Arsenic and compounds	1.9 μg/l	moderate	74.0	88.8	74.0
Benzo[a]pyrene	---	gap	-	-	-
1,3-Butadiene	---	gap	-	-	-
Chromium (hexavalent)	1.2 μg/l	moderate	12.9	-	-
TCDD	---	gap			
**Food and Beverages**					
Arsenic and compounds	varies by food	low	25.9	31.0	25.9
Benzene	varies by food	very low	4.4	10.0	2.4
Benzo[a]pyrene	varies by food	very low	2.2	0.4	1.4
1,3-Butadiene	varies by food	gap	-	-	-
Chromium (hexavalent)	varies by food	gap	-	-	-
TCDD	varies by food	gap			
**Indoor Dust**					
Arsenic and compounds	---	gap	-	-	-
Benzo[a]pyrene	2.91 μg/g	low	22.9	4.4	14.0
Chromium (hexavalent)	4.25 μg/g	very low	1.2	-	-
TCDD	---	gap			

1. CPF = cancer potency factor.

2. CA = California Office of Environmental Health Hazard Assessment.

3. Health Canada.

4. United States Environmental Protection Agency.

Substances that were negligible or not carcinogenic in a specific pathway were excluded.

Cancer potency factors for the same substance can differ by several orders of magnitude between agencies, due to interpretation of epidemiological and animal studies. We therefore present results using cancer potency factors derived by the California Office of Environmental Health Hazard Assessment (CA OEHHA) [38], Health Canada [13,21,39,40] and the United States Environmental Protection Agency (US EPA) [22,41].

## Results

**Figure 2:**
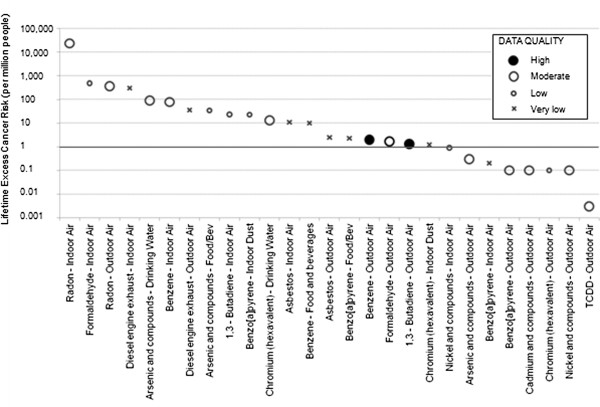
Estimates of lifetime excess cancer risk (per 1 million persons) ranked from highest to lowest for different environmental carcinogens and exposure pathways in Canada.

LECR indicators for 27 carcinogen-exposure pathway combinations were calculated for selected known carcinogens (Figure 2 and Table 2). Substances that are thought to make a negligible contribution to exposure or that are not carcinogenic in a specific pathway are excluded. Substances that might be important but where no data were available to make this determination are included to highlight data gaps. LECRs of between 1 and 10 per million due to non-occupational exposures are generally treated as being ‘essentially zero’ or ‘acceptable’ by a range of federal and provincial Canadian agencies [13]. Here we use 1 per million as a threshold for consideration to prioritize for additional assessment, given the screening nature of the approach. Substances with LECRs above 1 per million based on data of moderate to high quality may be targeted for more detailed risk assessments, such as those using probabilistic methods to better characterize the range of potential exposures given current measured levels. Similarly, substances with LECRs below 1 per million based on moderate or high data quality may not be important to prioritize for further study. Whenever data quality is assessed as low or very low, or no data were found, it may be useful to undertake additional research or monitoring to better characterize LECRs for comparative purposes.

### Indoor air

Based on average measured levels in Canada circa 2006 and the highest available cancer potency factor, potential LECRs are greater than 1 per million due to inhalation of asbestos, benzene, 1,3-butadiene, diesel engine exhaust, formaldehyde, and radon in indoor air. The risk posed by radon exposure (LECR of 23,655) is magnitudes larger than the next largest LECR (formaldehyde, with a LECR of 487). All have been calculated using an exposure frequency of 1, thereby implicitly assuming that each substance is always present in indoor air at the measured average level.

Data quality for measured concentrations of carcinogens in indoor air ranges from very low to moderate-high. The LECR associated with diesel engine exhaust is based on measured levels of total fine particulates by NAPS monitoring sites, and the assumption that 18 percent of total fine particulates are from diesel engine exhaust [42], and that 60 percent of ambient fine particulates infiltrate to indoor residential environments [43]. Data quality for the diesel engine exhaust estimate is therefore considered to be very low. For all other substances listed, concentration data were found exclusively in published literature and reports, as there are no national programs regularly monitoring indoor air quality in Canada. Moderate data quality ratings were assigned only to those substances for which consistent levels were reported in at least three reasonably comparable studies (benzene, formaldehyde, and radon). Low ratings were given to those substances with fewer studies available and/or some inconsistency in reported levels across studies (asbestos, 1,3-butadiene and nickel). Data gaps exist for arsenic, cadmium, hexavalent chromium, and 2,3,7,8-tetrachlorodibenzo[p]dioxin (TCDD).

We did not find enough publicly available data to develop regional indicators for indoor air. In general, indoor air quality is influenced by the varying uses of many consumer products, cooking practices, wood, candle and incense burning, and so on. We therefore expect more random variation among residences, rather than distinct regional differences due to these factors. Outdoor air quality, however, may also contribute to indoor air quality via infiltration through open doors, windows and gaps or cracks in building walls, and the regional patterns seen for outdoor air may be present in indoor air levels as well.

### Outdoor air

LECRs for a number of carcinogens in outdoor air circa 2006 are greater than 1 per million; however, they are much lower than those for indoor air when considering the same substances. In some cases this is due to lower outdoor ambient concentrations, but is also influenced by the much larger amount of time spent indoors over the average lifetime. In outdoor air, LECRs based on average concentrations and the highest available cancer potency factor are greater than 1 per million for asbestos, benzene, 1,3-butadiene, diesel engine exhaust, formaldehyde and radon. Radon and diesel engine exhaust pose substantially larger risks than the other carcinogens. Again, we used an exposure frequency of 1, thereby assuming that these carcinogens are present in all outdoor air. Data from the NAPS monitoring network suggests that this is a reasonable assumption. While we did not find much reported data for asbestos, several studies suggest it is ubiquitous in urban environments [44] (Refs here). Radon, however, is not present at significant levels in many regions of Canada, given the geological nature of the source. The use of an exposure frequency factor of 1 here should be interpreted as applying only to those who live in regions known to have potential for higher levels of radon.

A data quality rating of moderate was assigned to radon as the most recent outdoor measures are from a study conducted in 1990 in 17 Canadian cities [45]; we include them here although the data were gathered prior to 2000, as there is no expectation that outdoor levels would decline over time. Data quality for asbestos levels in outdoor air was considered to be very low. In Canada, only a few studies on outdoor asbestos levels were identified and those were conducted in communities impacted by asbestos mines. We used data from a US measurement program conducted in the 1980s and 1990s in the US intended to measure asbestos levels inside buildings thought to be contaminated with asbestos-containing materials [44]. Outdoor levels at each building were also measured to provide a comparison of indoor/outdoor concentrations, and we use the average of these for the LECR reported here.

The remainder of the carcinogen concentration data for outdoor air was estimated using NAPS monitoring data and ranges from very low to high based on the number of monitoring stations. A low rating was assigned to hexavalent chromium, as only total chromium is measured, and we used the assumption that 5 percent of total chromium measured is hexavalent [46]. Moderate ratings were assigned to substances with more than 10 monitoring locations across Canada (arsenic, benzo[a]pyrene, cadmium, formaldehyde, nickel and TCDD) and a high rating was assigned to benzene and 1,3-.butadiene, as they are measured at more than 50 locations across Canada. Data quality for diesel engine exhaust is considered to be very low for the reasons previously discussed.

Outdoor air quality can vary substantially both locally (i.e., within an urban area) and regionally (among different urban areas, and between urban and rural areas). The LECRs presented here are based on the annual average level for each carcinogen measured across monitoring stations in Canada. Geographic variation in LECRs associated with outdoor air is not reported here, but has been characterized by applying both statistical and deterministic models, as detailed in Hystad et al. (2010) [47] and on the CAREX Canada website, i.e., arsenic for example [48].

### Drinking water

Estimated LECRs are greater than 1 per million due to ingestion of arsenic and hexavalent chromium in drinking water. For arsenic, we expected levels to be higher in regions with greater abundance of naturally occurring arsenic outside of Ontario, and therefore used arsenic values reported in the Canadian Drinking Water Guideline Technical Document [49] and assigned a data quality rating of moderate. We used the assumption that all of the chromium present in drinking water is in hexavalent form [50], and data quality is judged to be moderate based on both the sample sizes ranging from approximately 259 to 329 for the year 2006 in DWSP [28], and the comprehensive review of data provided in the guideline document. For both arsenic and hexavalent chromium, we employed an exposure frequency of 1. In the case of arsenic, given the geological nature of the source, the indicator should be more carefully interpreted as applying to those Canadians living in regions where arsenic is known to be a drinking water contaminant.

No useful data for benzo[a]pyrene, 1,3-butadiene, or TCDD were identified. No cancer potency factors are reported by Health Canada, the US EPA, or the CA OEHHA for the ingestion of formaldehyde, nickel and cadmium.

We did not identify enough publicly available data to provide regional indictors for carcinogens in treated drinking water, in large part due to drinking water quality data being held by many thousands of local municipalities and not in a centralized national database. We also expect significant geographic variation of arsenic levels particularly when drinking water is drawn from private wells, based on geological sources of arsenic. Data that would support the development of regional indicators on drinking water quality in private wells were not publicly available.

### Food and beverages

Estimated LECRs are greater than 1 per million due to ingestion of arsenic, benzene and benzo[a]pyrene in foods and beverages, based on average concentrations and the highest available cancer potency factor. We were able to calculate exposure frequencies for each food, given the number of detections in each sample, and these were used to develop the average daily intake values. In practice, this means that for a given substance and food group (i.e., vegetables) the average daily intake estimate is influenced by the exposure frequencies of each of the included foods.

We assigned a data quality rating of very low or low to all relevant substances for which we found data. In general, Canadian data available in peer-reviewed studies, government reports or public online databases are limited in terms of consumption levels, foods tested, substances measured, the geographic representation, and temporal relevance. Typically, data were available only for a few of the 206 included food items. Table 3 summarizes the number of foods in each major group with data and the percent of total consumption represented by those with data. No suitable data were found for 1,3-butadiene, hexavalent chromium, or TCDD in prepared foods and beverages. No cancer potency factors are reported by Health Canada, the US EPA, or the CA OEHHA for the ingestion of formaldehyde, nickel and cadmium.

**Table 3:** Number of foods per food group with data and percent of total consumption represented

	**Food Groups**													
	**Meats/Oils**		**Seafood**		**Fruit**		**Vegetables**		**Dairy/Eggs**		**Grains**		**Beverages**	
	**(n = 11)**		**(n = 3)**		**(n = 50)**		**(n = 67)**		**(n = 28)**		**(n = 9)**		**(n = 18)**	
**Carcinogen**	(a)	(b)	(a)	(b)	(a)	(b)	(a)	(b)	(a)	(b)	(a)	(b)	(a)	(b)
Arsenic	6	46%	2	54%	8	24%	12	26%	3	13%	0	0%	1	1%
Benzene	4	52%	0	0%	11	38%	11	26%	8	20%	3	63%	5	21%
Benzo[a]pyrene	3	8%	2	54%	0	0%	1	2%	2	9%	0	0%	1	2%

(a) number of foods in food group with concentration data.

(b) percent of total consumption in food group represented by foods with concentration data.

We were unable to identify any publicly available data to support the calculation of regional LECRs for exposures via food and beverages. We do not expect significant regional variation in LECRs given the widespread geographical distribution of foods and beverages in general; however, there could be important differences for populations relying on locally grown and harvested foods.

### Indoor dust

Only four of the selected carcinogens are expected to be relevant via ingestion of indoor dust. Estimated LECRs are greater than 1 per million for benzo[a]pyrene and hexavalent chromium respectively, assuming average concentrations, maximum cancer potency factors and an exposure frequency of 1. Data quality is low for benzo[a]pyrene, and very low for hexavalent chromium. Typically, only one or two recent North American studies per substance were identified, limiting data representativeness. No useful data for arsenic or TCDD were identified. We were unable to include recent studies of indoor dust reporting only substance weight per area sampled (e.g., micrograms per cubic centimeter), because a concentration (e.g., grams per kilogram) is required to calculate LECR.

Geographic variation in LECRs for exposure via dust might be influenced by outdoor air concentrations due to industrial and vehicle emissions, as well as by indoor sources for some carcinogens (i.e., wood burning and cooking practices for benzo[a]pyrene). Data limitations severely hamper any effort to understand regional trends in exposures to benzo[a]pyrene, arsenic, hexavalent chromium, or TCDD via indoor dust.

## Discussion

We developed indicators of Canadians’ exposure to known carcinogens in the environment circa 2006, using existing and regularly collected environmental data and a risk-based approach, which are suitable for tracking population trends over time and help to prioritize exposure reduction activities. Known carcinogens with moderate to high levels of data quality and LECRs greater than 1 per million included: benzene and radon in outdoor air; benzene and radon in indoor air; and arsenic and hexavalent chromium in drinking water. The five highest lifetime excess cancer risks in Canada are associated with radon exposure indoors (LECR 23,655), formaldehyde exposure indoors (LECR 487), radon exposure outdoors (LECR 371), exposure to diesel engine exhaust indoors (LECR 300) and exposure to arsenic and compounds in drinking water (LECR 89). These five substances combined represent 99% of the total LECR estimated for all substances/exposure pathways, although data quality was low for radon in outdoor air and very low for diesel engine exhaust in indoor air. Other important data gaps were identified for asbestos and hexavalent chromium in both indoor and outdoor air, diesel exhaust in outdoor air, and in general for carcinogens dust, and food and beverages.

The LECR approach has been in use for several decades as a screening-level risk assessment tool. For example, using 24-hour personal exposure data collected as part of the Total Exposure Assessment Measurement (TEAM) studies conducted between 1980 and 1987 in 8 US cities, and cancer potency factors from the US EPA, Wallace (1991) reported a lifetime cancer risk of 120 per million for benzene in indoor and outdoor air combined [51]. Our estimate for Canadians, 20 years later, is approximately 22 per million. For the TEAM studies, the average level of benzene in indoor air was reported to be in the range of 7 micrograms per cubic meter (μg/m^3^), and 6 μg/m^3^ in outdoor air [52], while Canadian data circa 2006 suggest average levels in the range of 2.4 μg/m^3^ and 0.9 μg/m^3^ in indoor and outdoor air respectively, which is consistent with documented trends in benzene concentrations for outdoor air [24]. More recently, Logue et al. (2011) compiled data on measured levels of a number of hazardous air pollutants in indoor air of US residences and reported cancer risks in excess of 10 per million for formaldehyde, benzene and 1,3-butadiene, and cancer risks well below 10 per million for benzo[a]pyrene and nickel, which is consistent with our results [53]. In Europe, LECRs for benzene and formaldehyde in indoor air in various countries ranged from approximately 4 to 250 and from approximately 65 to 375 respectively [54], which is also broadly consistent with our results.

In outdoor air, McCarthy et al. (2009) analyzed ambient concentrations measured at US government monitoring stations from 2003 to 2005 inclusive [55]. They report LECRs between 1 and 10 per million for the median benzene and 1,3-butadiene concentrations, which is generally similar to our results, but report a higher LECR for median arsenic (closer to 10 per million, versus 0.1 to 0.3 per million based on mean of Canadian data) and a lower LECR for median formaldehyde (roughly between 0.01 and 0.1 per million versus 0.8 to 1.7 per million based on mean of Canadian data). Modelled outdoor air concentrations of toxic pollutants for each county in the US are used in the US EPA National Air Toxics Assessment (NATA) program [56]. For the year 2005, NATA reports average LECRs in the US similar to those reported here for inorganic arsenic (0.7 versus 0.3 per million), cadmium (0.07 versus 0.1 per million), nickel (0.08 versus 0.1 per million), benzene (3.3 versus 2.0 per million) and 1,3-butadiene (0.6 versus 1.3 per million). NATA also reports average LECRs for total chromium (0.56 per million) and total polycyclic aromatic hydrocarbons (0.8 per million), which are higher than the LECRs of 0.1 per million for hexavalent chromium and <0.1 for benzo[a]pyrene reported here. The average LECR reported for formaldehyde by NATA is a magnitude higher than our LECR (16 versus 1.7 per million).

Loh et al. (2007) calculated lifetime excess cancer risks for a number of airborne organic compounds using modeled distributions of concentrations in various microenvironments (outdoor and indoor at home, in offices, dining establishments, grocery and non-grocery commercial buildings, and during commuting) to develop estimates of representative total personal exposures [57]. Reported LECRs for benzene and 1,3-butadiene ranged from approximately 10 to 100 per million, compared to our LECRs of 12 to 80 per million and 4 to 25 per million respectively (including both outdoor and indoor estimates). The LECR for formaldehyde was lower (approximately 100 per million) in Loh et al. (2007) than that reported here (228 to 490 per million). This is likely due mostly to the difference in input values with Loh et al. (2007) using 18 μg/m^3^ to represent the geometric mean for indoor air in the US compared to our estimate of 33 μg/m^3^ representing the mean for indoor air in Canada. Both our estimate for benzo[a]pyrene and that of Loh et al. (2007) for polycyclic aromatic hydrocarbons (PAHs) as a group in air were less than 1 per million.

Few comparable LECR estimates for arsenic, benzo[a]pyrene and benzene in food and beverages were identified. Loh et al. (2007) also calculated LECRs for dioxin and PAHs via ingestion of food specifically [57]. We did not identify enough relevant data to estimate a LECR for dioxins (specifically TCDD) via ingestion of food, but our LECR estimate for benzo[a]pyrene specifically in foods ranges from less than 0.1 to 0.2 per million, far lower than the range provided in Loh et al. (2007) of approximately 10 to 50 per million for PAHs as a group.

We did not find many relevant and comparable studies for drinking water and dust exposure pathways. No current peer-reviewed studies reporting LECR estimates for arsenic in drinking water in North America were identified; however, the current Canadian Drinking Water Guideline for arsenic is 0.3 micrograms per litre (μg/L), which is also stated as being equivalent to a LECR of between 1.9 to 13.9 per million [49]. Our LECR for arsenic in drinking water is approximately 89 per million based on an average input concentration of 1.9 μg/L. Maertens et al. (2008) assessed the LECR associated with the ingestion of PAHs in settled house dust by preschool-aged children as being in the range of 1 to 100 per million [58]. The LECR reported here for ingestion of benzo[a]pyrene alone in dust, over a full lifetime, is 23 per million.

Of special interest are the LECR estimates for radon in indoor (23, 655) and outdoor air (371 per million). Inhalation via indoor air is well recognized as a key exposure pathway, and is estimated to be the second leading cause of lung cancer in Canada [59]. Although radon in homes has been measured extensively in Canada, we considered data quality to be moderate only, given that radon exposure follows geological patterns, and the substantial effect building type and condition can have on radon levels even in homes located next to each other. The average of the available data therefore represents those homes that have been measured, rather than what might be expected in all Canadian homes. Measured outdoor levels in Canada suggest this pathway may also be important. We found only one study, conducted in 1990/91, that measured radon in outdoor air near Canadian residences and reported 3 month average concentrations ranging from non-detection to as high as 118 Becquerels per cubic metre (Bq/m^3^) [44]. Although individuals move about when outdoors, time spent outdoor near their homes could be associated with significant exposures when radon is present. The long-term average time of the measured data (3 months) suggests that although radon is dissipated in outdoor air, potential exposure levels can remain high enough to be of concern, even if indoor exposures are decreased.

In general, differences between our estimates and others using the same methodology would arise solely from the use of different parameter inputs (concentrations, cancer potency factors, or population characteristics). It is therefore critical that all parameter inputs are clearly documented, enabling others to assess their comparability and validity. The use of a standard approach however, provides internal consistency and supports direct comparisons across exposure pathways and between substances for screening level purposes.

Importantly, variability among individuals exists, both in terms of exposure levels and responses to those exposures. For any individual, exposure will vary both temporally (short and long term) and spatially depending on a multitude of factors (proximity to carcinogen sources, behaviours affecting intake rates, etc.). There is also clear evidence that exposures during key lifestages may be more important than at other times, particularly during childhood and even pre-natally, and more specific cancer potency factors may be required to better reflect these susceptibilities [60,61]. For these reasons, the LECRs presented here are best used as general relative indicators, and should not be interpreted as real cancer risks or estimates of future disease burden.

Uncertainty in our indicators also exists, and is influenced not only by potential measurement error in the concentration data, but also by the use of short duration samples to represent long term concentrations; comparability of concentrations across studies when different data collection methods are used; the use of small samples (potentially non-random) to represent larger populations; and the use of data from limited geographic regions to represent national concentrations.

This significant lack of nationally representative data (both geographically and temporally) does not allow us to establish the prevalence of exposure in the Canadian population, and may impede identifying trends over time if new data do not become available. In addition, establishing trends in future updates of the indicators may be difficult, particularly for those that depend solely on data from published literature or government reports, as the number of new studies undertaken that specifically measure environmental concentrations may be small, and/or changes in the LECR estimates may represent enhanced data rather than actual trends in exposure. Improvements in analytical methods may also affect how often substances are detected and at what levels, and therefore the resulting indicators. The authors plan to undertake a recalculation of the indicators presented here using data representative of conditions in 2011 when available, the results of which will provide further insight into the feasibility of regular updating and ease of comparability across time periods.

This study suggests there are real opportunities to improve our understanding of Canadians’ exposures to carcinogens through undertaking more population-representative national monitoring programs. These would produce better estimates of average levels, the probable distribution of exposure levels throughout our population, and therefore more effectively targeted prevention programs. These types of programs are likely more feasible in government or government partnership settings rather than solely academic.

In lieu of undertaking probabilistic methods for exploring variability and/or uncertainty in the input values (due to limited data availability), we developed a simple database tool (eRISK); available from the authors or via the CAREX Canada website on request [62]. The tool can be used to examine the range of daily intakes and associated risks for any number of scenarios. For example, users can input values that might better represent the range of regional conditions (minimum, average or maximum concentration) or the unique dietary intakes of different population groups, as well as adjust the standard lifestage parameters and cancer potency factors.

This paper describes only one aspect of the CAREX Canada environmental project. Other components focus on providing the same indicators for selected suspected carcinogens (IARC Group 2A and 2B); identifying geographic variation in environmental concentrations and risk; standardizing and ranking carcinogen emissions by different geographical areas in Canada; and reviewing existing food consumption and residue databases in Canada.

## Conclusions

The risk-based approach provides a flexible method for developing comparable, substance-specific estimates of lifetime daily average intake and associated LECRs for a variety of exposure pathways, including outdoor air, indoor air, drinking water, dust, soil, and foods and beverages, using available data. The indicators do not represent real cancer risk for any individual; however, they do identify what the LECRs are if environmental concentrations remain unchanged over time. If environmental concentrations increase or decline in the future, so will the LECRs. Most important, perhaps, is the usefulness of this standardized risk assessment-based approach for comparative risk assessment and for identifying data quality issues and data gaps, which serves to highlight where future efforts should be targeted to improve our understanding of Canadians’ exposures to carcinogens.

## Abbreviations

Bq/m^3^: Becquerels per cubic meter; CA OEHHA: California office of environmental health hazard assessment; CPAC: Canadian partnership against cancer; CR: Concentration – response; DWSP: Ontario drinking water surveillance program; EPI: Exposure potency index; f/ml: Fibres per millilitre; IARC: International agency for research on cancer; LECR: Lifetime excess cancer risk; NAPS: National air pollution surveillance; NATA: National air toxics assessment; PAH: Polycyclic aromatic hydrocarbon; TCDD: 2,3,7,8-tetrachlorodibenzo[p]dioxin; TEAM: Total exposure assessment measurement; US EPA: United states environmental protection agency; US: United States; μg/m^3^: Micrograms per cubic meter; μg/L: Micrograms per liter.

## Competing interests

The authors declare that they have no competing interests.

## Authors’ contributions

ES developed and led the overall study, conducted data reviews and analysis, and prepared the manuscript. PH participated in data review and analysis, and collaborated in the preparation of the manuscript. KP, RC, and ACL participated in data review and analysis. PAD provided advice during the study development and manuscript preparation. All authors read and approved the final version.
